# Rodents as vehicle for delivery of transgenic bacteria to make paratransgenic sand fly vectors of cutaneous leishmaniasis in field condition

**DOI:** 10.1038/s41598-023-41526-2

**Published:** 2023-09-09

**Authors:** Marzieh Ghassemi, Amir Ahmad Akhavan, Alireza Zahraei-Ramazani, Bagher Yakhchali, Mohammad Hossein Arandian, Reza Jafari, Maryam Akhlaghi, Leila Shirani-Bidabadi, Kamal Azam, Mona Koosha, Mohammad Ali Oshaghi

**Affiliations:** 1 Department of Vector Biology and Control of Diseases, School of Public Health, Tehran University of Medical Sciences, Tehran, Islamic Republic of Iran; 2 Institute of Industrial and Environmental Biotechnology, National Institute of Genetic Engineering and Biotechnology (NIGEB), Tehran, Islamic Republic of Iran; 3 Isfahan Health Research Station, School of Public Health, Tehran University of Medical Sciences, Tehran, Islamic Republic of Iran; 4 Department of Vector Biology and Control, Faculty of Public Health, Kerman University of Medical Sciences, Kerman, Islamic Republic of Iran; 5 Department of Biostatistics, School of Public Health, Tehran University of Medical Sciences, Tehran, Islamic Republic of Iran

**Keywords:** Microbiology, Molecular biology, Diseases

## Abstract

Vector-borne diseases, among them leishmaniasis, cause more than 700,000 deaths annually. The lack of an effective vaccination and the increasing resistance of sand flies to insecticides require the urgent development of innovative approaches to contain the disease. The use of engineered bacteria that express anti-parasite molecules (paratransgenesis) shows much promise. However, a challenge for implementation of this strategy is to devise means to introduce modified bacteria into sand flies in the field. In this study, we use rodent food bait as a delivery strategy to introduce two mCherry-fluorescent bacteria, *Serratia* AS1 and *Enterobacter cloacae*, into adult sand flies in field settings. Bacteria-infected food was provided to *Rhombomys opimus* rodents. These bacteria transiently pass through the rodent alimentary tract and are delivered to larval habitats with the rodent feces. The feces are ingested by sand fly larvae and, in the case of *Serratia* AS1, are trans-stadially transmitted to adults. This is the first report of targeting delivery of *Serratia* AS1 in a paratransgenic system to control transmission of leishmaniasis under field condition. This novel strategy shows promise for delivering transgenic bacteria to *Leishmania* vectors in the field.

## Introduction

Leishmaniasis is caused by parasites of the genus *Leishmania*. These parasites are transmitted by the bite of female phlebotomine sand flies of the genus *Phlebotomus* in the Old World and *Lutzomyia* in the New World^1,2^. Three basic manifestations of the illness are cutaneous, mucocutaneous, and visceral. Skin ulcers are characteristic of the cutaneous form, whereas mouth, nose, and skin ulcers are characteristics of the mucocutaneous type. The visceral form manifests by fever, low red blood cell count, and enlarged spleen and liver. Skin symptoms may occur after the initial visceral leishmaniasis in the form of Post Kala-azar dermal leishmaniasis (PKDL). More than 20 different species of *Leishmania* are responsible for human infections^2–4^.

A total of 97 out of 200 nations and territories are endemic for leishmaniasis. Leishmaniasis is widespread, from deserts of western Asia and the Middle East to the rainforests of Central and South America. With 700,000 to 1 million new cases annually, it may affect as many as 12 million people worldwide^3,4^.

Human leishmaniasis has two main means of transmission: (i) from animal reservoirs in case of zoonotic^1,5^ and (ii) transmission from person to person in case of anthroponotic leishmaniasis^1,6,7^. Wild or domestic animals, such as hyrax, dogs, foxes, wolves, and rodents may serve as reservoirs for zoonotic leishmaniasis.

Because of the complexity of biological and epidemiological conditions of transmission, that includes human or animal reservoirs, a variety of parasites and sand fly vectors, disease control requires a combination of strategies^1,5,8^. Developing vector insecticide resistance and parasite resistance to drugs are hampering the fight of parasite infection and treatment. These limitations point to the need for the development of novel and more effective strategies that impact minimum environmental effects^3,9–11^.

Paratransgenesis, consisting of engineering symbionts to produce anti-pathogen molecules, shows promise for combating pathogen transmission by insect vectors. Some examples are listed in Table 1**.** Of note, paratransgenesis is compatible with other control methods^39^. One of the most important challenges in paratransgenesis is to find a robust delivery system to introduce the engineered symbiont to vectors in the field. In endemic foci of ZCL in Iran, the *Leishmania*-carrying sand flies prefer to lay their eggs in reservoir rodent dwellings. Sand flies larvae feed on rodent’s feces in their nests, in depths of 1–2 m below the earth’s surface. We hypothesized that if these feces were contaminated with bacteria, they could be transmitted to larvae, and in case of *Serratia* As1, be further transmitted from larvae to adults^40^.

**Table 1:** Microbes with potential use for paratransgenic control of *Plasmodium*, arboviruses, *Trypanosoma* and *Leishmania* spp.

Original microbe	Insect vector	Key references
*Asaia*	*Anopheles stephensi*	^12–14^
	*Anopheles gambiae*	^15^
*Escherichia coli*	*Anopheles stephensi*	^14^
	*Rhodnius prolixus*	^16^
*Enterobacter cloacae*	*Anopheles stephensi*	^17^
	*Phlebotomus papatasi*	^18^
*Pantoea agglomerans*	*Anopheles stephensi*	^19^
	*Anopheles gambiae*	^19^
*Serratia* AS1	*Anopheles gambiae*	^20–23^
	*Anopheles stephensi*	
	*Culex pipiens*	
	*Culex quinquefaciatus*	
	*Culex theileri*	
*Metarhizium anisopliae*	*Anopheles gambiae*	^24^
AgDNV*	*Anopheles gambiae*	^25^
*Rhodococcus rhodnii*	*Rhodnius prolixus*	^26–29^
*Corynebacterium sp.*	*Triatoma infestans*	^30,31^
*Sodalis glossinidius*	*Glossina sp.*	^32–36^
*Bacillus subtilis*	*Phlebotomus argentipes*	^37,38^

*AgDNV* : Anopheles gambiae* densonucleosis viruses.

Recently, two bacteria, *Serratia* AS1 and *Enterobacter cloacae* have been evaluated as paratransgenesis candidates against vector-born disease^18,20–23,41,42^. In this study we tested rodent food bait containing *Serratia* AS1 or *E. cloacae* as a delivery method to adult sand flies, using laboratory and field conditions.

## Materials and methods

### Study area

**Figure 1:**
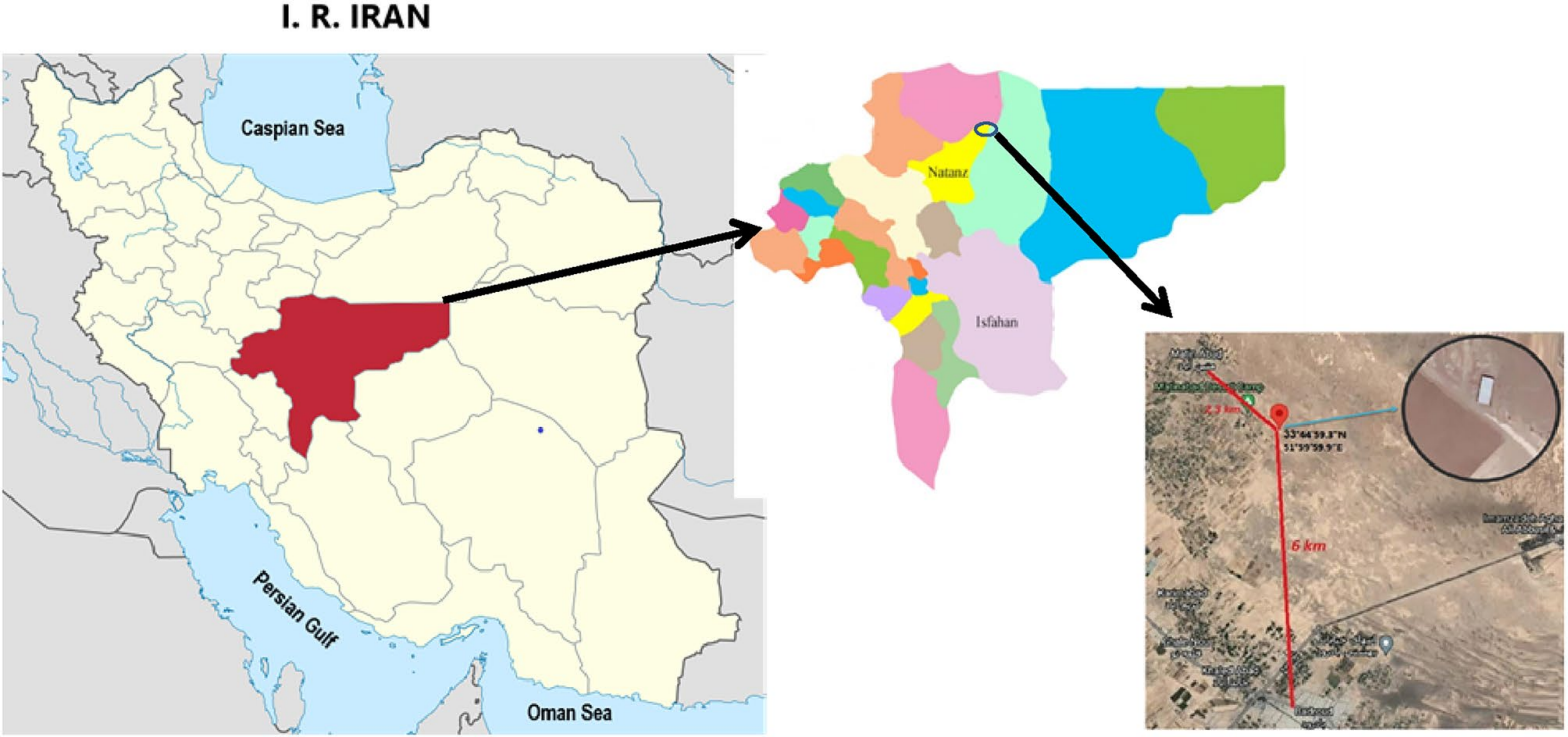
Location of the semi-field enclosure. Exact geographical coordinates and aerial photo of the vivarium are shown. It is located about 6 km north of Badroud City, Isfahan Province and is 2.3 km to the closest village.

Our research was conducted on a site that will henceforth be referred to as the “field site”, located in the Isfahan Province at 33° 44′ 59.3′′ N 51° 59′ 59.9′′ E with altitude of 981 m above sea level. The province is known as one of the most important leishmaniasis foci, in the country. It is located about 6 km north of Badroud City and is 2.3 km to the closest village (Fig. 1). *Phlebotomus papatasi* is the dominant and the main sand fly vector species in this region. Rodents used for experiments were collected in this location. Sherman traps were used to capture alive *Rhombomys opimus* (Muridae: Gerbillinae) which is the dominant and the most important reservoir in the region.

### Establishment of transgenic bacteria expressing mCherry fluorescent protein

*Serratia* AS1-mCherry a facultative aerobic, Gram-negative, rod-shaped bacterium belonging to the Enterobacteriaceae family was a gift from Prof. Jacobs-Lorena from the Department of Molecular Microbiology and Immunology, Malaria Research Institute, Johns Hopkins Bloomberg School of Public Health. It was generated by integration of a pBAM2 plasmid carrying a mCherry fluorescent protein gene^23,41^. The plasmid was constructed by replacing a beta-lactamase (ApR) gene of the plasmid pBAM1 with expression cassette^43^. pBAM2-mCherry was transformed into both bacteria (*Serratia* AS1 and *E. cloacae*) using heat shock method^44^. *Enterobacter cloacae* subsp*. dissolvens*, a rod-shaped, gram-negative bacterium belonging to the family Enterobacteriaceae was isolated from microflora of *Ph. papatasi* from a zoonotic cutaneous leishmaniasis area in Isfahan, central Iran^45^. The transformed bacteria were cultured on Luria–Bertani (LB) agar plate containing the apramycin (80 µg/ml) antibiotics for about 16 h, after that single colony of mCherry-fluorescent bacteria was isolated and cultured on LB agar at 28 and 37 °C for *Serratia* AS1 and *E. cloacae* respectively. Stocks of bacteria were prepared and stored in – 80 °C for further use. In all the lab and field experiments, besides observation of fluorescence protein, a selective LB medium containing the apramycin (80 µg/ml) antibiotics was used thoroughly. The transformed bacteria and sand fly specimens fed on the transformed bacteria were used as a positive and the wild type bacteria and wild sand flies not fed on the transformed bacteria were used as negative controls.

### Preparing bacterial suspension

The transformed bacteria were cultured in LB broth at 28 °C for *Serratia* and 37 °C for *E. cloacae* in shaking incubator until reaching OD_600_ = 1 (10^9^ cell per ml), then cells were harvested by centrifugation (3000×*g*, 10 min), washed twice in sterile phosphate-buffered saline (PBS), and resuspended in 5% (w/v) sterile sucrose solution to obtain 10^9^ cells/ml. The bacterial suspensions were used for our experiments.

### Gastrointestinal transit time in *R. opimus*

To perform the test of whether the bacteria can pass through rodents alimentary and its speed, nine female, non-pregnant, 200–300 g weight of *R. opimus* (great gerbil) were selected randomly from the animal unit in Isfahan Health Research Station, and divided into 3 groups of three rodents each. There were no exclusion criteria except sex. The first and second groups were offered food infected by the modified *E. cloacae* subsp*. dissolvens* and *Serratia* AS1 expressing cherry-fluorescent respectively, and the third group was used as control. A mix of 10 g pellets (parsfeed®, consisting of wheat straw, sodium chloride, maize, fish meal, whey powder, soybean dehulled, dicalcium phosphate, soybean oil, corn, calcium carbonate and yeasts) and 10 g carrots (as a source of water) were coated with 1 ml bacteria suspension (10^9^ cell per ml suspension per gram of food) and given to the treatment groups; the same amount of food without bacteria was available to the control group. Rodents were then transferred to disinfected solitary cages. Their fecal samples were collected every 2 h and 0.1 g of each fecal sample was homogenized in 900 µL of PBS, serial dilutions were plated on LB agar medium and then fluorescent colonies were counted. The whole process was repeated 3 times. The sixth author was aware of the group allocation, and then the rest of experiment was followed blindly by first author. All the animals were healthy and normal at the end of the experiment.

### Stability of bacteria in *R. opimus* feces

Rodent stool samples that were positive for transgenic bacteria were divided into two parts and stored at 24 °C to evaluate bacterial stability. One part was stored in 1.5 ml plastic closed-lid micro tube and another part in open-lid micro tube. Bacteria were assayed daily using serial dilution on LB agar medium and CFUs were counted.

### Stability of bacteria in *R. opimus* food

Experiments were conducted to determine bacteria stability when added to rodent food in laboratory (26 °C, 25% RH) and field conditions (Minimum temperature: 39.5 °C and Maximum 51 °C, with 11.5–19.6% RH). Colony-forming units (CFUs) in rodent food containing 10^9^ bacteria/gram of food were determined by daily sampling for 12 consecutive days. Sampling was carried out as follows: 0.1 g food was removed and was added to 900 µl of sterile PBS buffer and serially diluted. A total of 100 µl was cultured in LB agar plates and bacteria CFUs per gram of food were calculated.

### Vivarium setup

**Figure 2:**
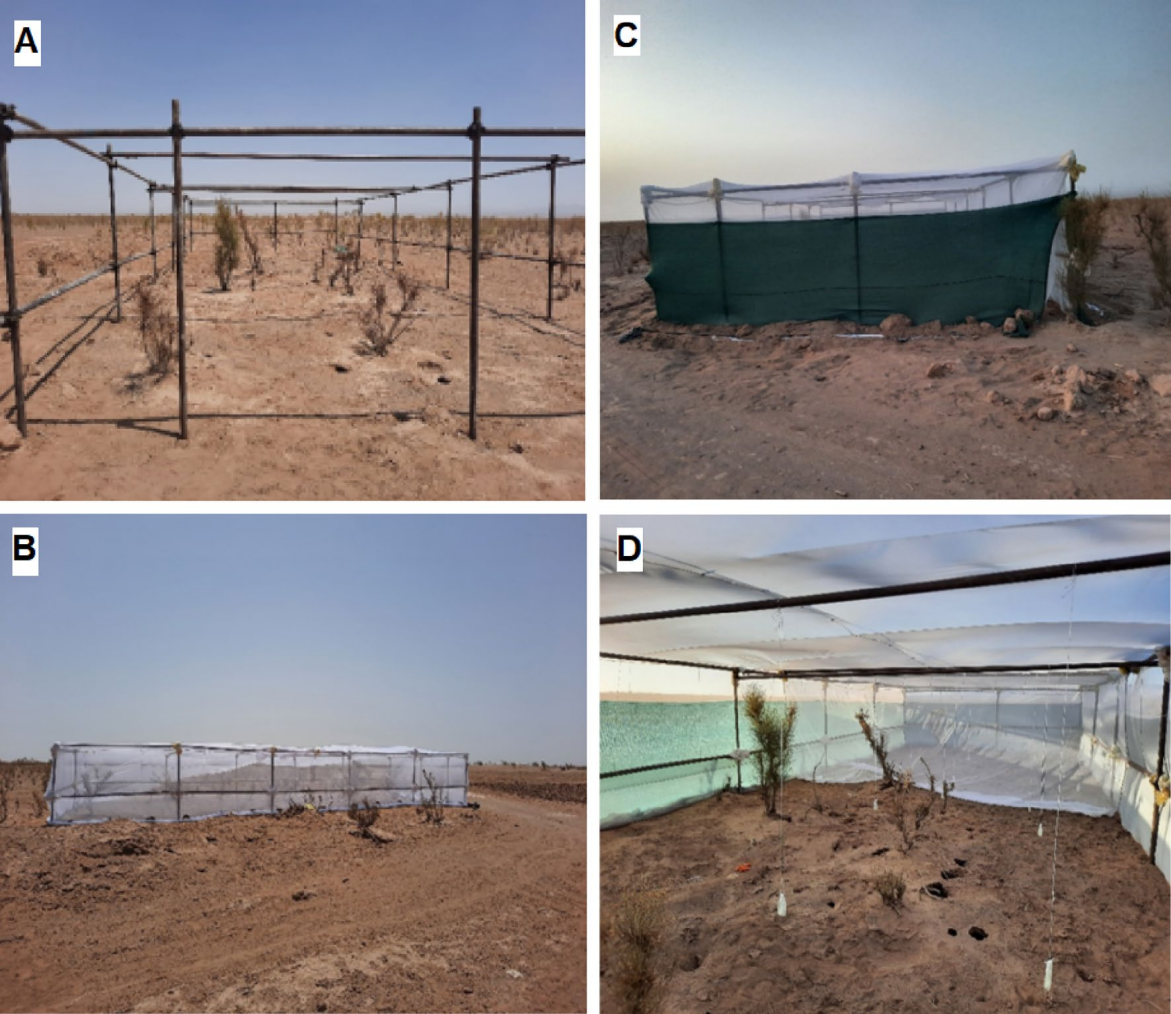
Vivarium design. (**A**) Framework was installed using scaffolds measuring 15 × 6 × 2.5 m. (**B**) The framework was fully covered with a mosquito net with very fine mesh to prevent sand fly escape. (**C**) Green shade net was used all around the vivarium to protect against wild animals. (**D**) Iinterior view of vivarium.

The semi-field enclosure (hereafter, vivarium) was built using scaffold frameworks measuring 15 × 6 × 2.5 m. The framework was fully covered with fine-mesh (400 holes/cm^2^) cotton mosquito net to prevent sand fly escape. The interior was divided into two equal parts accessible via zipper doors, each part for one of the bacteria. For protecting the net from wild animals, green plastic shade net was used all around the enclosure (Fig. 2). The vivarium's position was carefully chosen to include naturally populated by high numbers of wild sand flies, three separate wild *R. opimus* colonies, and at least five shrubs of the area's dominant vegetation, *Haloxylon persicum*, on either side of it. Using mosquito net allows the vivarium to have the exact same temperature, sunlight and air circulation of the surrounding environment.

### Adult sand fly bacterial acquisition via larval diet

#### Laboratory tests

For conducting this section of our test, using a hand aspirator, live sand flies were captured and transferred to sand fly insectary of School of Public Health, Tehran University of Medical Sciences. Sand flies were fed on the blood of BALB/c mice. After feeding blood-engorged females with few males were moved to oviposition pots which were lined with plaster of Paris and covered with fine mesh net. They were fed with honey solution (50%) and saturated sucrose. After oviposition and death of adult sand flies, the remnants were removed, and pots were maintained at 26 ± 2 °C and 72 ± 10% relative humidity. Pots were checked daily for hatched eggs. The larvae were fed by powdered diet consisting of rabbit food (pellet) and rabbit excrement^46^ laced with the two bacteria (1ml suspension containing 10^9^ cells per 1 gr larval food). Adult sand flies which emerged from these pots were divided into three groups (1) intact specimens without sugar or blood meal (2) fed on sugar meal and (3) fed on both sugar and blood of BALB/c mouse. After 24 h the sand flies were chilled to death, homogenized, and cultivated on LB Agar media after being surface sterilization with 70% alcohol. The specimens were washed by PBS and the washout was used as negative control to ensure that bacteria were not getting transmitted mechanically.

#### Field tests

As mentioned, chambers were set up where rodents and sand flies naturally occur. Bacteria were prepared the same way as for laboratory tests. The food consisting of pellets and carrots coated with bacteria (1ml suspension containing 10^9^ cells per gram of food), were provided to rodents in a separate chamber for each bacterium. Wheat (50 g) was added to the food as a mean to prevent rodents to leave the vivarium, as this is their main food source in natural conditions. About 20 g of fresh food contaminated with the bacteria were supplied for each rodent burrow every day. Ten days after starting to feed the rodents on the bacteria, sand flies that emerged from larvae that fed on the rodent feces, were captured at regular interval every three to four days and transferred into individual cages. The flies were frozen 24 h after blood feeding on mice and after being surface cleaned with PBS and sterilized with 70% alcohol, they were homogenized in 100 µl PBS and plated on LB agar media. To confirm sand fly’s surface sterilization, the captured sand flies were washed with PBS and then the washed PBS was plated for the fluorescent bacteria, and in case of infection to the bacteria, that specimen was discarded from analysis.

### Statistics

Significance of bacteria relative abundance between samples was analyzed using the Mann–Whitney U test. Means were compared using Student’s *t* test. SPSS version 27 was used for all statistics. *p* < 0.05 was considered statistically significant.

### Ethics approval

The protocols were conducted in this study followed the guidelines of the institutional ethical committee (School of Public Health & Allied Medical Sciences- Tehran University of Medical Sciences, SPH-TUMS). The protocols were approved by Biomedical Research Ethics Committee of SPH-TUMS under registry IR.TUMS.SPH.REC.1399.243. We confirm the study is reported here was performed in accordance with ARRIVE guidelines.

## Results

We tested the introduction of fluorescent *Serratia* As1 and *E. cloacae* subsp. *dissolvens* into adult sand flies by feeding rodents with food containing the bacteria and exposing sand fly larvae to their feces.

### Recording of gastrointestinal transit time in *R. opimus*

**Figure 3:**
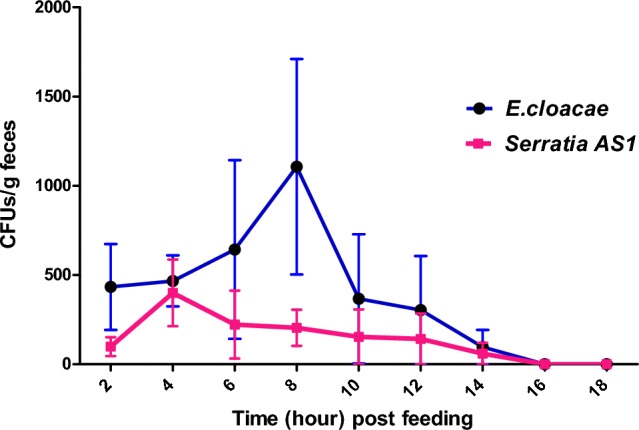
Time course of gastrointestinal transit (consumption to excretion) for *Serratia* AS1 and *Enterobacter cloacae* expressing cherry fluorescent protein after feeding *Rhombomys* *opimus* with bacteria-laced food. Colony-forming units (CFUs) of feces suspension plated on LB agar culture medium are shown. Numbers of bacteria are means of three replicates (mean ± SEM). There were no statistically significant differences between the two groups (*Mann*–*Whitney U test****,*** *p* = 0.662).

Fluorescent *Serratia* As1 and *E. cloacae dissolvens* were fed to *R. opimus* and bacteria number in their feces was measured at different times. The peak number for *Serratia* AS1 bacteria was at 4 h after bacteria feeding, while for *E. cloacae* bacteria peaked at 8 h after feeding (Fig. 3). No bacteria were found after 14 h.

### Survival of bacteria in close/open lid

**Figure 4:**
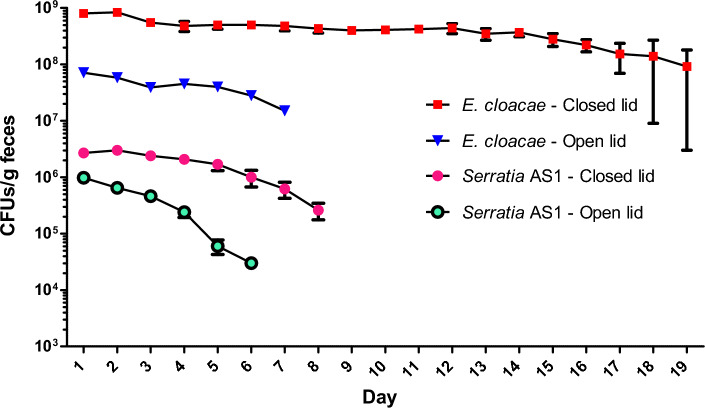
*Serratia* AS1 and *Enterobacter cloacae* survival in *Rhombomys* *opimus*’s feces in micro tubes with closed and open lid*.* There were statistically significant differences between the two bacteria and the two conditions (*Mann*–*Whitney U test,* *p* < 0.001). Numbers of bacteria are means of three replicates for each bacterium (mean ± SEM). With exception for *E. cloacae*-close lid, no bacteria were found after the last time points shown in the figure.

This test was conducted to mimic natural situation of faeces and their bacteria in the depth of rodent burrows as close lid and ground surface as open lid. We found that both bacteria are more stable under a closed lid environment. *Serratia* AS1 is less stable than *E. cloacae*, and the latter can be detected in cultures for a longer period of time (open lid: 19 versus 8, close lid: 7 versus 6 days) (Fig. 4). Also, the abundance of *E. cloacae* (e.g., at maximum point with 9.3 × 10^8^ CFUs/g feces) was several folds higher than *Serratia* AS1 (at maximum point 3 × 10^6^ CFUs/g feces).

### Survival of bacteria on *R. opimus*’s diet

**Figure 5:**
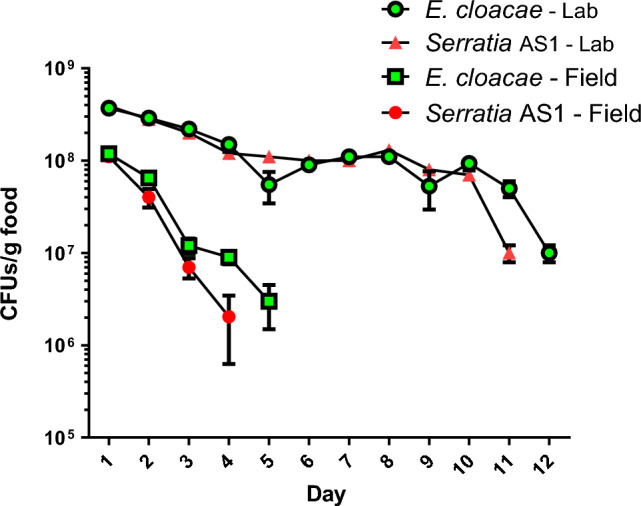
Survival of *Serratia* AS1 and *Enterobacter cloacae* in *Rhombomys* *opimus’s* food in the laboratory (26 °C, 25% relative humidity) and in the field. In the field, the average daily minimum and maximum temperatures for the period of the experiment were 39.5 and 45 °C respectively, with a range of 11.5–19.6% relative humidity*.* There are no statistically significant differences between the two bacteria in each condition (*Mann*–*Whitney U test,* *p* = 0.542 and *p* = 0.460 for field and lab conditions respectively). However, the difference between lab and field conditions for each bacterium is statistically significant (*Mann*–*Whitney U test, p* < *0.001).* Numbers of bacteria are means of three replicates for each bacterium (mean ± SEM).

Both bacteria were detected in the food up to day 12 in the laboratory and only up to day 4 in the field (Fig. 5). No bacteria were found after the last time points shown in the figure.

### Transstadial transmission

#### Laboratory tests

Sand fly larvae were fed with a powdered diet, consisting of rabbit food and rabbit excrement, laced with the two bacteria. A total of 50 emerged adult sand flies (20 males and 30 females) were tested for transstadial transmission of *Serratia* AS1 from the larvae. We found that six out of 20 males (30%) harbored 3–7 CFUs respectively. Out of the 30 females, five were tested immediately after emergence, without sugar feeding, and were negative for *Serratia* AS1-mCherry. The 25 remaining females were fed on sugar and 18 of them succeeded to feed on blood. Three out of seven sugar-fed female flies were positive for *Serratia* AS1-mCherry with 16–27 CFUs. Eleven out of the 18 blood-fed sand flies were positive for *Serratia* AS1-mCherry, with 1100 to 2150 CFUs colonies at 24 h post blood feeding. Altogether, 20 out of 50 (40.0%) of sand flies were positive for the bacterium in laboratory experiments. As for *E. cloacae*, we found only one positive fly (out of 50 tested) carrying 226 CFUs 24 h post blood feeding.

#### Field tests

**Figure 6:**
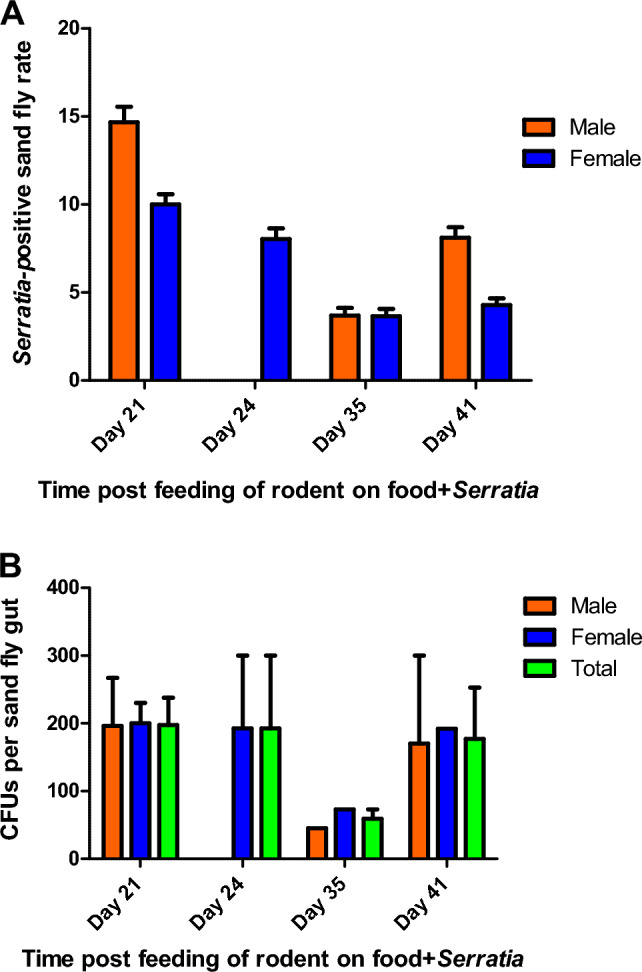
Presence of bacteria in adult *Ph. papatasi* sand flies emerged from field vivariums occupied by rodents that were fed with fluorescent bacteria. (**A**) Rate of adult sand flies acquired *Serratia* AS1 bacterium from the larvae that fed on *Rhombomys opimus* feces. The animals fed on food impregnated with *Serratia* AS1-mCherry bacteria. Totally 12 (12.4%) adult sand flies out of 97 captured sand flies were revealed positive for *Serratia* AS1*-*mCherry from day 21 to 41 (6 males 6 females). (**B**) Mean (± SEM) number of *Serratia* AS1-mCherry CFUs in the captured adult sand flies 24h post blood meal.

Ten days after starting to feed the rodents on the bacteria, sand flies that emerged from the rodent burrows were captured at regular interval every three-four days and transferred to individual cages. A total of 515 *Ph. papatasi* adult flies were captured from two vivariums. From one vivarium containing three rodent colonies (each colony contains about 7 animals and was considered as one replicate) that were fed with *E. cloacae*, 249 sand flies (87 males, 162 females) were captured; from the other vivarium containing three rodent colonies that were fed with *Serratia* AS1, 266 sand flies (108 males, 158 females) were captured. Sampling was performed at regular (3–4 days) interval. Of the 418 (out of 515) flies that were caught during the first 20 days, none was positive for either of the two bacteria. Sand flies positive for *Serratia* AS1 first appeared on day 21 post feeding of the rodents with food containing the modified bacteria. Additional positive flies were also found on days 24, 35, and 41. A total of 12 (six males and six females) out of 97 (12.4%) of the sand flies captured from day 21 to day 41 were positive for the *Serratia* AS1-mCherry bacterium (Fig. 6A). The numbers of the bacterium colonies in the gut of sand flies ranged from 40 to 300 CFUs per specimen with a mean of 167.9 CFUs (Fig. 6B). Only one female sand fly specimen that had been captured on day 24 was infected with *E. cloacae*-mCherry and contained 10 CFUs. These results indicate that *Serratia* AS1can be transferred transstadially in *Ph. papatasi* sand flies in natural conditions, while *E. cloacae* transfer is very rare.

## Discussion

We showed that *Serratia* AS1-mCherry can be introduced to *Ph. papatasi* larvae and then to adults via *R. opimus* feces as a source of infection. The *Serratia* added to the wild *R. opimus* food (carrot or pellet) passes through the rodent digestive tract and is then excreted with the feces in the hidden nests where sand fly larvae breed. The temperature and RH in the depth of nest is respectively much cooler and higher than outside of rodent burrows which are in favor of bacterial survival, as we showed that high temperature and low RH have a big effect on the survival of bacteria on rodent food. The bacteria ingested by the larvae are then transstadially transmitted to the pupae and adult sand flies. We found that, in adult sand flies carrying transgenic bacteria expressing defensin, the development of *Leishmania* parasites is inhibited^18^. Transstadial transmission of *Serratia* AS1 has also been reported in *An. gambiae, An. stephensi*, and *Culex pipiens* mosquitoes^21–23^.

We found that *Serratia* AS1 can be introduced to both adult male and female sand flies. It has been shown that some bacteria such as *Serratia* AS1 can be transmitted horizontally during copulation (venereal transmission)^20,23,47–50^, and we have previously shown that *Serratia* AS1 is transmitted venereal from *Ph. papatasi* males to females^51^. Venereal transmission of *Serratia* AS1 facilitates introduction of the bacteria into sand fly populations. In addition, it provides potential use of non-vector and harmless male sand flies as a delivery route for genetically modified *Serratia* AS1 producing anti-*Leishmania* effector molecules. We note that *Serratia* AS1 can be transmitted for three *An. stephensi* generations^20^; it will be of interest to determine for how many sand fly generations this bacterium can persist.

Sand flies harboring *Serratia* first appeared 21 days post-feeding of rodents on food baits containing the fluorescent bacteria. From days 10 through 18, 98 adult sand flies were captured, none of which were positive for the bacteria. The first positive flies were observed on day 21. This time gap (first 20 days) agrees with the sand fly life cycle, which takes about 20 days for development from third/forth instar larvae to adults^52,53^. Flies that were positive on days 35 or 41 post-feeding of rodent on food baits probably represent the smaller larvae (1st–2nd stage) which needed more time to develop to adults.

We investigated the stability of the two modified bacteria when added to the rodent food under field and laboratory conditions. We found that there is a sharp decline in bacterial numbers of both *E. cloacae* and *Serratia* AS1 on rodent food, but that a considerable number of viable bacteria (more than 200,000 CFUs per ml of food suspension for both bacteria) remained until day four under field conditions. However, no bacteria were found after four days, indicating that it is necessary to replace food baits with fresh baits every four days. This loss may be caused, at least in part, by the loss of the plasmid encoding the fluorescent protein, as was found to be the case for *Anopheles* mosquitoes^20^.

We have conducted an open/closed lid experiment to mimic the natural situation of feces and their bacteria in the depth of rodent burrows as close lid and ground surface as open lid. Although, this experiment in some way mimics the natural situation particularly in case of temperature and RH, however, a more detailed and precise experiment is needed to measure the environment parameters such as oxygen concentration (or air composition), humidity, and temperature in these two different filed environments.

In this study, the bacteria were administered to the rodents via food (pellet and carrots), which is high in fiber, is an easy and practical delivery system, and is like the *R. opimus* diet in nature. Moreover, we observed that the two bacteria cannot colonize the *R. opimus* alimentary canal and only transiently pass through it, being egested with the feces. We detected both bacteria in the feces for 14h after feeding, with a peak at 4 h for *Serratia* AS1, and 8h for *E. cloacae.* This agrees with the findings of Padmanabhan et al.^54^ who showed that it takes 6–8 h for activated charcoal to reach the colon and fecal pellet. The mean number of excreted bacteria for those peak hours was 311,000 bacteria (CFU) for *Serratia* AS1 and 874,000 for *E. cloacae*.

Adult sand flies may pick up bacteria from at least one of the following three sources: plant saps during sugar feeding, animal host skin while taking a blood meal, or transstadially, where bacteria are transmitted from larvae to adult^55–57^. For the paratransgenesis approach against pathogens, understanding bacterial acquisition routes is crucial, since it indicates how best to introduce transgenic bacteria to vectors in field conditions. In this study, we found that *E. cloacae* could not be transmitted transstadially to adult sand flies. Although it has been shown that wild or modified *E. cloacae* has the potential to be used in control of insects^58,59^ or to block parasite transmission in mosquitoes and sand fly vectors^18,42,43^, this bacterium cannot be transmitted via the transstadial route^17^. Thus, alternative delivery of this bacterium needs to be explored, using other routes, including sugar baits and animal host skin^23,58,59^.

In this study we observed the negative result of bacteria detection in adults immediately after emergence, prior to the intake of sugar or blood. It is known that the quantity of bacteria pass from larvae to adult stage will diminish strongly and reach to few to no bacteria in the midgut during metamorphosis because huge physiological and biochemical variations, involving the larval immune system and loss of the peritrophic membrane due to the midgut enzymes’ activities^41,60–65^.

Safety and non-pathogenic of the modified bacteria to other organisms including humans, animals, and harmless or beneficial insects are some important prerequisites before releasing the transformed bacteria in nature. The requirements for using engineered bacteria for paratransgenesis have been reviewed by Ratcliffe et al. 2022^66^. There are some studies assuring some of these requirements for safety of *Serratia* AS1 before released into the environment. For example, Huang et al.^20^ showed lack of horizontal gene transfer in *An. stephensi* infected with fluorescent *Serratia*. These authors demonstrated a self-limiting event for the modified *Serratia* because the bacteria had lost the plasmids used for transforming returning to the wild type after three generations of mosquitoes. However, concerns about the possible pathogenicity of the modified *Serratia* towards animals and humans must be considered since there is a report on opportunistic pathogenicity of *Serratia* in humans^67^, even though the pathogenic bacteria belonging to *S. marcescens* which is not the same as the *Serratia* bacteria isolated from insect^41^ and used in this study.

In this study we showed that under the desert conditions in summertime when the temperature was maximum and RH was minimum in the vivarium, 12% of emerging adults were infected. We suggest that if the bacteria apply from the beginning of sand fly seasonal activity and repeated throughout the spring, summer, and early autumn, this rate will be increased since the environment condition in spring and autumn is milder and support the bacteria survival. This method can be used to control ZCL in Iran and neighboring countries with the same or similar rodent reservoir hosts and vector that share the same microhabitat, where infected food can be easily delivered. These reservoir rodents are reported in central and south Asia from Uzbekistan, Kyrgyzstan, Kazakhstan, Turkmenistan, Tajikistan, Pakistan, Iran, Afghanistan, to southern Mongolia, and north-western China^68–76^. However, it would be challenging to apply this method to more complex or less well-known transmission cycles, e.g., in tropical forests of South and Central America where different wild and synanthropic rodents are reservoirs of American cutaneous leishmaniasis (ACL)^77^.

**Figure 7:**
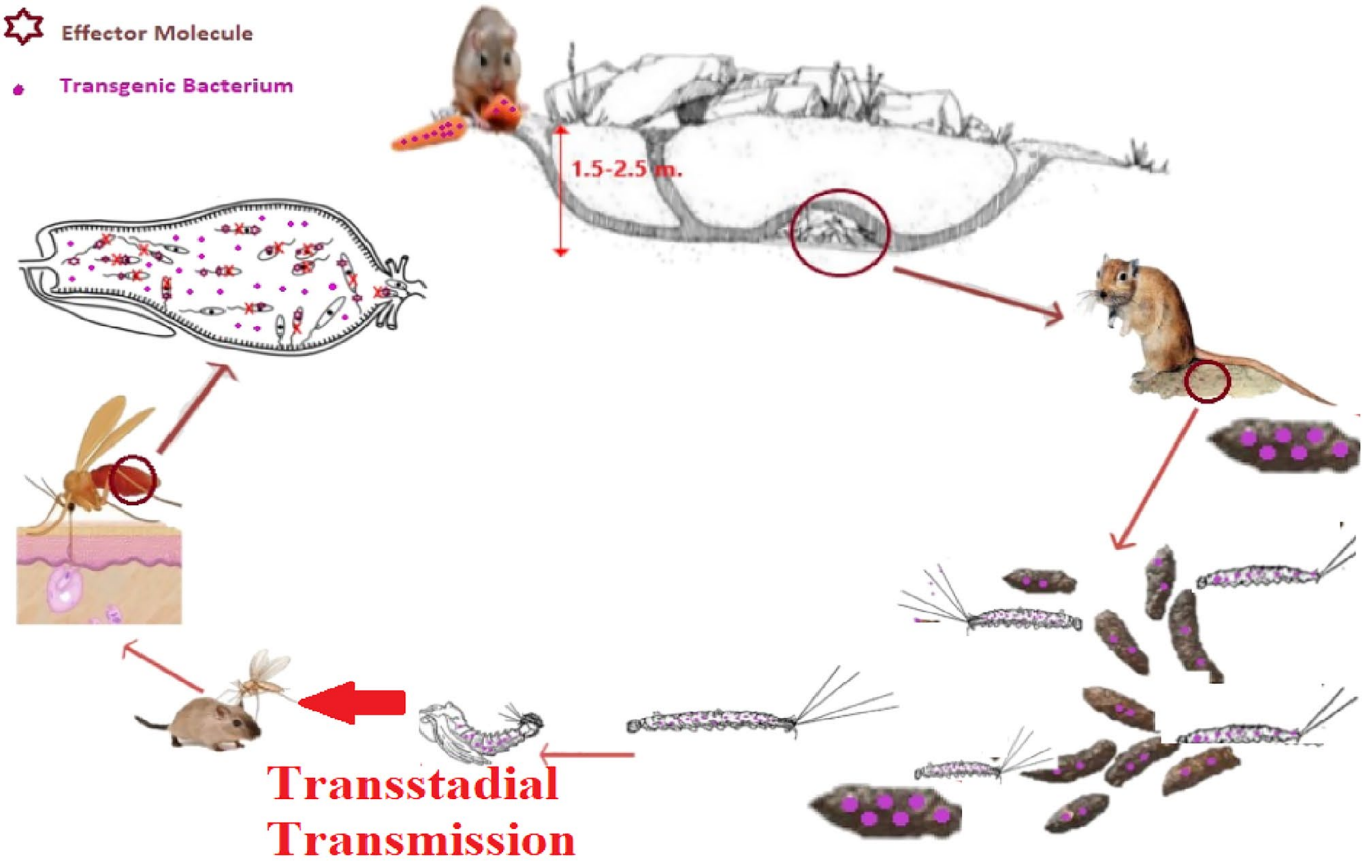
Rodents as vehicle for delivery of transgenic *Serratia* AS1 bacteria to *Phlebotomus papatasi* sand fly vectors of cutaneous leishmaniasis in field condition.

In summary, we show that transgenic *Serratia* AS1 bacteria can be introduced into sand flies utilizing rodents as carrier, a delivery strategy that may be used in the field (Fig. 7). Other delivery systems that directly target adult sand flies, including sugar baits and animal host skin may complement this approach.

## Data Availability

All the data are available and presented in the text.
